# Humanization of Antibodies using a Statistical Inference Approach

**DOI:** 10.1038/s41598-018-32986-y

**Published:** 2018-10-04

**Authors:** Alejandro Clavero-Álvarez, Tomas Di Mambro, Sergio Perez-Gaviro, Mauro Magnani, Pierpaolo Bruscolini

**Affiliations:** 1 Departamento de Física Teórica, Universidad de Zaragoza, Zaragoza, 50009 Spain; 2 Department of Biomolecular Sciences, University of Urbino “Carlo Bo”, Urbino, Italy; 3 Centro Universitario de la Defensa, Zaragoza, 50090 Spain; 4 Instituto de Biocomputación y Física de Sistemas Complejos (BIFI), Universidad de Zaragoza, Zaragoza, 50018 Spain

## Abstract

Antibody humanization is a key step in the preclinical phase of the development of therapeutic antibodies, originally developed and tested in non-human models (most typically, in mouse). The standard technique of Complementarity-Determining Regions (CDR) grafting into human Framework Regions of germline sequences has some important drawbacks, in that the resulting sequences often need further back-mutations to ensure functionality and/or stability. Here we propose a new method to characterize the statistical distribution of the sequences of the variable regions of human antibodies, that takes into account phenotypical correlations between pairs of residues, both within and between chains. We define a “humanness score” of a sequence, comparing its performance in distinguishing human from murine sequences, with that of some alternative scores in the literature. We also compare the score with the experimental immunogenicity of clinically used antibodies. Finally, we use the humanness score as an optimization function and perform a search in the sequence space, starting from different murine sequences and keeping the CDR regions unchanged. Our results show that our humanness score outperforms other methods in sequence classification, and the optimization protocol is able to generate humanized sequences that are recognized as human by standard homology modelling tools.

## Introduction

Antibody-based drugs have acquired an increasing importance in the last two decades, both for imaging and for therapeutic uses, especially to treat different types of cancer and autoimmune diseases. However, their development is a long and difficult process, prone to fail at different stages. Antibody humanization is a key step in this process, unless the candidate is already obtained from a human library, and is essential in moving from the preclinical to clinical stage. In fact, new antibodies are typically developed in animal models (most often, in mouse); however, the antibodies obtained by this way are usually not tolerated by humans, eliciting *in vivo* an immune response against the murine antibody. Thus, they need to be “humanized”, substituting part of their sequences by the human ones, while preserving their specificity, affinity and stability. Although computational methods are available, nowadays such humanization process is mostly a trial-and-error process, based on CDR-grafting and back mutations^1^. CDR-grafting implies selecting the Complementarity-Determining Regions (CDRs), responsible for antigen recognition, from the given murine sequence and grafting them into the human Framework Region (FR); the latter is selected by looking, in the human genome, at the germlines that produce FRs most homologous to the murine ones: the hope is that the combination of such human frameworks with the original murine CDR will result in a molecule that still preserves its stability and activity, but is tolerated by the human immune system. However, most of the times this approach is not completely successful at either of its quests, and the researcher is left alone in trying further mutations, until an antibody with the selected properties is identified. This error-prone process is a true bottleneck in the development of new treatments, in a market of increasing global impact.

From an algorithmic point of view, CDR-grafting corresponds to a search, in the human germlines, for the sequence with minimal Hamming Distance (i.e highest similarity) to the original murine one. Thus, the similarity to the closest germline represents a “humanness score” to maximize, for this approach. More in general, any humanization protocol will rely on maximizing some “humanness score”, whose basic requirement is to be able to distinguish human from mouse sequences with as few errors as possible. Most humanness scores are based on pairwise sequence identity between the sample and a set of reference (most often, germline) human sequences: for instance, the score can correspond to the average similarity^2^, or the average among the top 20 sequences^3^, or the highest similarity, over “windows“ of typically 9 residues^4,5^. Recently, a different approach as been proposed by Seeliger^6^, introducing a score function that accounts both for local preferences and for pair correlations between residues at different positions. Interestingly, such approach is reasonably capable to distinguish between human and mouse sequences, despite a relevant residual overlap between their distribution; also, the stochastic humanization process under such score function samples regions of low immunogenicity, even though the final basin of attraction of the trajectories presents an intermediate value of immunogenicity (as measured by the Epivax score)^7^. It is also worth mentioning that such approach breaks with the common logic of considering CDRs as the only antigen binding regions, by treating correlations between any positions (CDR or framework) on the same grounds, which can be a safer option due to the fact that there are relevant antigen binding residues also in framework regions^8^. However, Seeliger’s approach uses an ad hoc score function for the pairs of residues, $\mathrm{ln}\,({p}_{ij}^{2}({A}_{i},\,{A}_{j})/({p}_{i}({A}_{i}){p}_{j}({A}_{j}))$, that apparently is loosely related to mutual information $M{I}_{ij}={\sum }_{{A}_{i},{A}_{j}}\,{p}_{ij}({A}_{i},{A}_{j})\,\mathrm{ln}({p}_{ij}({A}_{i},{A}_{j})/({p}_{i}({A}_{i}){p}_{j}({A}_{j}))$, and may suffer its same problems^9^, in distinguishing direct and indirect correlations. A more fundamental approach deals with the observed sequences as instances of a general probability distribution *p*(**A**) over the sequences ${\bf{A}}=({A}_{1},\,\ldots ,\,{A}_{L})$. By constraining the sequences according to the observed site *f*_*i*_(*A*_*i*_) and pair *f*_*i,j*_(*A*_*i*_, *A*_*j*_) frequencies, and looking for the distribution maximizing the Shannon entropy, one finds $p({\bf{A}})={e}^{-H({\bf{A}})}/Z$, where: $H({\bf{A}})=-\,{\sum }_{i < j}{e}_{ij}({A}_{i},{A}_{j})-{\sum }_{i=1}^{L}{h}_{i}({A}_{i})$ and $Z={\sum }_{{\bf{A}}}{e}^{-H({\bf{A}})}$. This is appealing, since it paves the way for a connection to statistical physics: *p*(**A**) appears as a Boltzmann distribution, corresponding to the energy function *H*, made up of a one-body term *h*_*i*_(*A*_*i*_), stating the preferences of each position for each amino acid type, plus an interaction term *e*_*ij*_, coupling the position *i* and *j*. These effective interactions should be regarded as the expression of the different constraints that intervene in the accelerated sequence evolution that immunoglobulins undergo during maturation, as for instance, the optimization of the interaction with the antigen, the need to preserve stability of the folded structure against unfolding or misfolding and aggregation, the requirement of low affinity for the T-cell receptors, to avoid an immunogenic response. The knowledge of the parameters *e*_*ij*_(*A*_*i*_, *A*_*j*_), *h*_*i*_(*A*_*i*_) above allows to calculate the probability of any sequence, opening the possibility to associate to each sequence a measure of its “humanness”. However, the inference of the parameters is a formidable task, that cannot be accomplished in an exact way. Techniques as Direct Coupling Analysis^9^ have been used to provide approximate estimates; here instead, we follow the approach by Baldassi and coworkers^10^ specifying a simple, quadratic form for the energy function *H*, that allows an analytic derivation of the parameters and of the posterior probability of any sequence. This approach has been recently used to predict antigen-antibody affinity^11^, with a different choice of the regularization, and using the quadratic form as a sequence score; here we elaborate on the original approach, and derive a score that is related directly to the posterior probability of a sequence, as explained below. We will still refer to this model as the “Multivariate Gaussian” Model (MG), even if the posterior probability score that we calculate and use is actually a multivariate Student distribution. We use this score to assess its efficiency in classifying murine and human sequences, comparing the method with simpler approaches just based on the Hamming Distance (i.e. sequence pairwise difference) between the input sequence and the human or mouse reference database. To obtain the latter, we curated human and murine learning and test database, of matching VH and VL chains, in order to assess the joint role of the light and heavy region in determining humanness.

Our goal is to understand the role of correlations between mutations at different positions, including across heavy and light chains, as accounted for by the statistical model, in determining the traits of humanness. Also, we want to understand whether the VH and VL chains behave and vary independently, as assumed implicitely by all humanization methods that deal separately with the two chains, or if correlations between the VH and VL chains play a relevant role, and cannot be neglected. Moreover we consider the relationship between our humanness score and the observed immunogenicity of a small set of antibodies for which the immunogenic reaction in patients has been reported in the literature. Finally, resorting to Steepest-Descent/Monte-Carlo simulations with the MG statistical score, we perform the humanization of a few murine sequences that have also been humanized experimentally, to see how our results compare with the experimental ones. We conclude by discussing the possible applications and future lines of research stemming from this approach. Before proceeding, let us mention that alternative strategies, to define a humanness score based on sequence distributions, could be attempted: for instance, one could resort to Hidden Markov Model (HMM) techniques to learn a probability profile from the alignment of the sequence database. However, such an approach does not appear to be very appealing, for our goals: indeed, Seeliger’s results^6^ suggest that correlations between pairs of residues are relevant. HMMs are effective in accounting local correlations between matching columns or neighboring sites (i.e. correlations that can be encompassed by the transition matrix from the state at position *i* to that at position *i* + 1 in the model graph), but are not suitable to describe generic long range correlations^12^, as those between VH and VL, whose relevance we want to assess. This does not rule out that alternative scores based on HMM could be effectively introduced, but we do not adopt this strategy here, and leave the subject to future investigations.

## Results

### Correlated and uncorrelated classification of test databases

**Figure 1:**
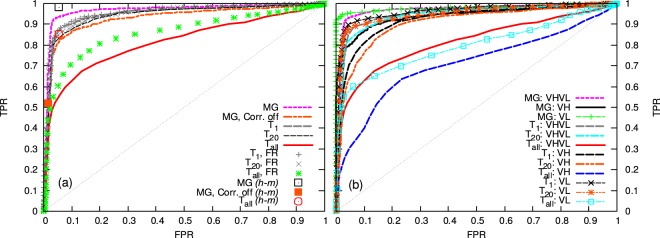
ROC curves for the different models. ROC curves obtained upon classification of the test human and murine databases, using the human learning database to learn the MG distribution, and to calculate the average distance for the “*T*_*all*_” and “*T*_*n*_” methods. Panel (a): ROC curves obtained using the full VHVL chain for classification, with or without CDR regions. Panel (b): ROC curves obtained using the VH or VL chain separately for classification. “Corr. off” indicates that correlations between residues have been removed in the MG model, “FR” refers to the curve obtained removing the CDRs and keeping just the framework regions, “h-m” indicates that classification is performed using both human and murine learning datasets as reference (see Methods).

After creating human and murine, learning and test databases of matching VH, VL regions, and fixing the parameters of the statistical model as described in Methods, we compare the performance of the distance based approaches with the statistical distribution method (Fig. 1), in distinguishing human from non-human (in our case, murine) sequences in the test database. Our goal is to assess if and to what extent the MG approach, accounting for two-sites correlation, improves the classification based on sequence-similarity criteria. We can see that the MG model efficiently distinguishes between murine and human sequences in the test database, scoring better than the distance-based approaches. Notice also that correlations do matter: as already pointed out by Asti and coworkers^11^ in a similar context, setting to zero the non-local interactions, and keeping only a block-diagonal distribution for the correlations (see Methods), results in a much worse performance. Interestingly, if we just eliminate correlations between the VH and VL regions, keeping those within the same chain, the performance is practically unaffected (data not shown): this supports the commonly used approach to deal with heavy and light chains separately.

Distance-based methods similar to the one proposed by Adler^3^, considering only the distance to the *n* closest sequences to the query one in the learning database, provides a better classification than accounting for the distance from the whole set of human learning sequences^2^ (the “*T*_*all*_” method). In fact, the ROC curves steadily improve as we move from *T*_*all*_ to *T*_1_, suggesting that the local structure of the sequence space may be more relevant than the global one, and that the heterogeneity in the human database can be misleading for a distance-based classification.

As it could be expected, upon removing the CDRs and keeping only the framework regions, the predictions by *T*_*n*_ improve (especially, for increasing *n*), supporting that the CDRs do not carry any relevant species-specific information. For the sake of completeness, it is also interesting to notice that using two different reference learning databases improves the classification performance of the MG model, but much more so that of the *T*_*all*_ approach: classifying a sequence according to whether its distance score to the human learning dataset is better than that from the murine learning dataset is very fast and efficient. However, this approach cannot be safely adopted for humanization, where the goal is to make a sequence “sufficiently human”, and not necessarily “as close to human and far from murine as possible”.

Classification based on just the VH or VL regions suggests that the VL region allows to better distinguish human from murine sequences: for the MG case, VL-based classification performs even better than the VHVL-based one, while VH based classification does not reach the same results. The same is true also for the *T*_*n*_ method. Thus, apparently the VL carries a greater amount of information on the human or murine nature of the sequence. Table S3 in Supplementary Information (SI) reports single-value indicators of the quality of the classification of the test databases with the different methods, complementing the information reported in Fig. 1.

### Classification of test, murine, chimeric, humanized and fully human antibodies

We classify the test database and the engineered antibodies according to the methods above, using the VHVL distribution, since this is the one that we will use later on for sequence design. We use the human learning distribution as the reference one for classification, and we fix the threshold score, separating human sequences from the rest, according to the value that maximizes the Youden’s index for the test database (see Methods; in general, the values maximizing the latter and Matthews Correlation Coefficient almost coincide, ensuring that the choice of a particular indicator is not crucial).

**Figure 2:**
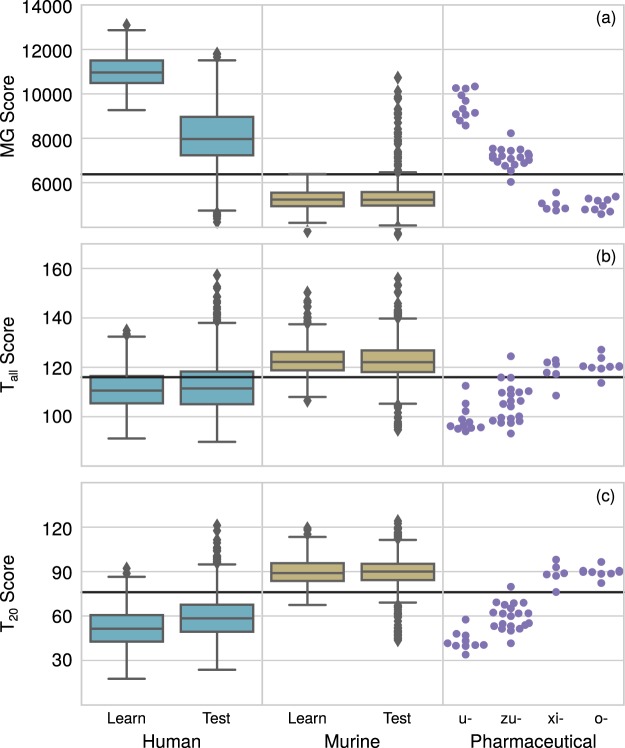
Boxplot of three score distributions for the different datasets: Panel (a): MG score; Panel (b): *T*_*all*_ score; Panel (c): *T*_20_ score. Pharmaceutical antibodies are indicated according to the suffix in their International Nonproprietary Name: “umab” are fully human antibodies; “zumab” are humanized antibodies, usually containing murine CDRs grafted on top of human framework variable regions; “ximab” are chimeric antibodies, obtained by assembling the whole murine variable region on top of a human constant part; “omab” are murine antibodies. Since we deal with just the antibodies’ variable regions, “ximab” and “omab” are indistinguishable. The horizontal lines signal the threshold score above (for the MG case) or below (for the other cases) which the sequence is classified as human. The threshold values are ${t}_{MG}=6383$, ${t}_{{T}_{all}}=116$, ${t}_{{T}_{20}}=76$.

Figure 2 reports the distributions of the scores for the learning and test databases, as well as for the pharmaceutic antibodies whose sequences are publicly available^13–15^ (see Methods and SI Table S1), labeled by the suffix of their International Nonproprietary Name (INN). Notice that the MG-scores of the human learning datasets are much higher than those of the human test dataset (and actually quite close to the maximum possible, *MG*_*max*_ = 13124.49). This is most likely a signal of overfitting of the learning dataset, which is inevitable due to the dimension of the matrix Σ and the number of aligned sequences. We expect that, as new researches allow to increase the size of the learning database, this issue would become less evident. Table 1 reports the fraction of correct predictions (for the therapeutic antibodies, we consider correct a prediction of -umabs and -zumabs as human, -omabs and -ximabs as murine).

**Table 1:** Fraction of correct predictions.

	Human	Murine	umab	zumab	ximab	omab
MG	1289/1388	1324/1379	11/11	19/20	6/6	9/9
*T*_1_	1225/1388	1296/1379	11/11	19/20	6/6	9/9
*T*_20_	1195/1388	1274/1379	11/11	19/20	6/6	9/9
*T*_*all*_	935/1388	1193/1379	11/11	19/20	5/6	8/9

Fraction of correct predictions for the test and therapeutic databases, using the threshold obtained as specified in Methods, to distinguish between human and murine sequences.

It can be noticed that MG yields the best results, with the highest fraction of correct classifications for the human and murine test databases; the performance of all the method in classifying therapeutic antibodies is similar, and almost perfect, for all methods, even if in that case the number of sequences is too small to be statistically relevant.

### Correlation of the MG score with immunogenicity

**Figure 3:**
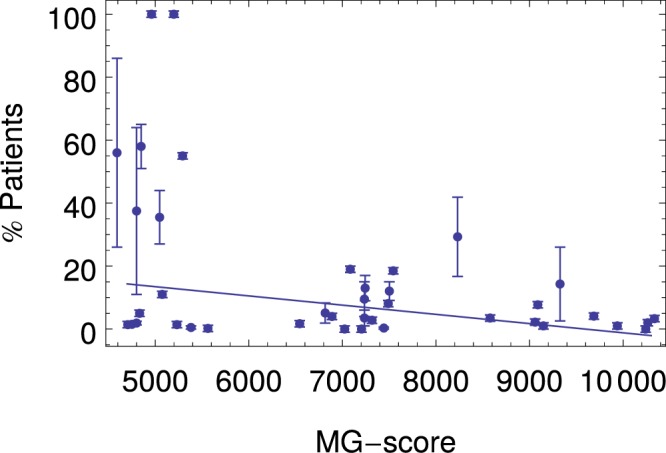
Scatter plot of the experimental immunogenicity and the MG score. The immunogenicity (% of patients that develop antibodies against the therapeutic antibody) is plotted versus the MG-score.

A good performance in classification tasks is the fundamental requirement for a humanness score, but it could be not enough if we want to use such score for antibody humanization. The reason is that sequences with better score might not be better sequences, since the MG score has been derived from sequence information only, and it does not explicitly account for e.g. protein stability, solubility, or immunogenicity. In particular, the latter is a crucial aspect for humanization, since it is highly desirable that a drug does not elicit an immune response. In the following we focus on the MG-score, as the best candidate to use in a humanization protocol. In order to estimate how good is the MG humanness score for humanization tasks, we compare it with the reported fraction of observed immunogenic response (as measured by the appearance of anti-drug antibodies) for several pharmaceutical sequences: Fig. 3 reports the correlations between the MG score and the experimental findings. Notably, clinical essays often report several values for the same drug, since immunogenicity appears to depend on the disease the drug is used for, as well as on the possible combination of the drug with other treatments. A little arbitrarily we have used the mean between minimal and maximal reported values as the average, and the semidifference between them as the error bar of each “measure” in the plot (when just one value is reported, an error of 1% is assumed). Then we have performed a linear fit of the experimental immunogenicity (with uniform weights), reported in the figure. As it can be seen, the experimental values are widely spread, especially at the murine end; this implies that immunogenicity is just loosely related to the degree of humanness of the variable regions. Hence, as expected, the fit *y* = 56.35 − 0.005806 *x* (with x the MG-score and y the experimental immunogenicity) is quite bad, with a low value of the “explained variance” *R*^2^ = 0.18, and a Pearson correlation coefficient of *C* = −0.43. However, the fit reveals a global trend of improvement in immunogenicity as the humanness score increases from murine to fully human antibodies.

The limited number of sequences for which an experimental estimation of immunogenicity exists, made us look for an alternative, in-silico definition, that we could apply to any proposed sequence. To this end, we have built an immunogenic score resorting to the MHCII software^16,17^ as described in SI Methods, predicting T-cell receptor epitopes in any given sequence, as a proxy for its immunogenicity: our goal was to compare the MG humanness with the immunogenic score for a broader set of sequences, and to test for both scores during the humanization process. However, the results, reported in SI Figs S2 and S3 do not indicate that such immunogenic score is sufficiently reliable, since its correlation with experimental data is even worse than that of the MG-score. Thus, we did not proceed further on this line.

### Statistical optimization of murine sequences

Next, we choose in the literature seven pairs of murine-humanized sequences (SI Table S2) and perform Steepest Descent (SD) and Simulated Annealing Monte Carlo (SAMC) simulations starting from the murine sequences, to obtain the sequences with the best MG-score. In all cases, we keep the CDRs fixed, since thet are associated to the antigen-recognition function that we want to preserve. We use two different algorithms due to their different nature: in the SD protocol, at each step we make the point mutation that most improves the score. The output is the closest local optimum to the original murine sequence. On the other hand, the SAMC procedure allows a more extensive exploration of the sequence space, and is less prone to get trapped in local minima, while looking for the global optimum.

**Figure 4:**
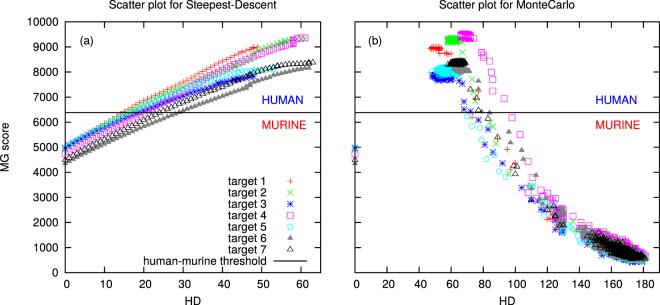
Scatter *MG score-Hamming distance* plot for the Steepest Descent (Panel (a)) and the SAMC (Panel (b)) for all our targets. Here HD is the Hamming distance between our proposed sequence and the original murine one. The SAMC trajectories start at the murine sequences, on the left (HD = 0, MG-score around 4500), then jump immediately to the region at the bottom-right of high HD, low score (highly non-human sequences, but very different from the original murine). Notice that all trajectories roughly overlap in this region: there is no memory of their different, and fixed, CDR sequences, and we witness a basically free exploration of the sequence space. Then, when the temperature falls below a certain threshold, the trajectories move to the top-left region, of highly human sequences with score and HD depending on the fixed CDR regions of the original sequence.

Figure 4 reports the MG score versus the Hamming distance to the original murine sequence, starting from each of the murine targets in SI Table S2, while a typical time-course for SD and SAMC simulations is reported in SI Fig. S4 for the first murine target. SI Fig. S4 shows that the MG score steadily increases with time in SD simulations, and the distance to the original murine sequence increases as well, which reinforces our previous findings that the MG score is a good measure of “humanness”. This correlation between time and HD from the original sequence results in monotonic curves for SD simulations in Fig. 4, from the original murine sequence on the left, to the final humanized one on the right. On the other hand, the distance to the experimentally humanized sequence in SI Fig. S4 does not present a monotonic decrease and does not reach zero, as expected: the experimentally humanized sequence need not be the only possible one, or even the “most humanlike” one at given CDRs. However, it is interesting to notice that, for all sequences, many of the proposed mutations coincide with those appearing in the experimentally humanized sequence (see column 7 in Table 2). In general, the SD final sequences contain more mutations than the experimental humanizations, suggesting that it is probably unnecessary to fully optimize the MG score, and that viable sequences can be found earlier in the optimization process, whose last steps involve usually smaller changes in the score. Notably, in all cases the MG-score of the experimentally humanized sequence is higher than the original murine one, again confirming the goodness of the MG-score as a humanness score; moreover, the score of the optimal SD sequence is higher than that of the experimental sequence. Another interesting comparison between SD predicted and experimental humanized sequences is shown in Table S4 in SI, where we report the result of a protein-BLAST search for the most similar sequence. Interestingly, the experimentally humanized sequence is not always recognized as human by BLAST, while for the SD sequence, always a human or humanized sequence appears as the most similar one, which again supports the goodness of the MG-score as a humanness score.

**Table 2:** Comparison between original, simulated and experimentally humanized sequences.

tgt	MG (o)	MG (h)	MG (p)	HD (p, o)	HD (h, o)	TP	FP	TN	FN	FPR	TPR	YJS
1	4963	7571	9000	49	37	31	18	246	3	0.07	0.84	0.78
2	4905	7184	9328	61	48	43	18	234	3	0.07	0.90	0.83
3	4976	6780	7817	48	37	24	24	241	9	0.10	0.65	0.57
4	4734	5689	9362	61	38	22	39	228	9	0.15	0.58	0.46
5	4978	7202	8131	53	39	37	16	243	2	0.06	0.95	0.89
6	4350	5193	8159	62	29	18	44	232	4	0.16	0.62	0.48
7	4481	5468	8379	63	35	24	39	229	6	0.15	0.69	0.56

Column 2, 3 and 4 report the MG-score for the original murine sequence “o”, the experimentally humanized sequence “h”, and the one predicted by SD “p”, for each target (column 1). Column 5, 6 report the number of mutations between pairs of sequences (HD). We define as “positive” (P) the mutations of the predicted sequence with respect to the murine one: P = HD (p, o); “negative” (N) the number of corresponding identical residues in the predicted and murine sequence. Accordingly, True Positive (TP) will be the number of common mutations, with respect to “o”, shared by “p” and “h”; False Positive (FP) indicates that in the predicted sequence there is a mutation with respect to the murine, but such mutation is not the present in “h” (or it is not the same mutation); True Negative (TN) imply that neither the predicted nor the humanized sequence have mutations, while False Negative (FN) indicates that the humanized sequence present a mutation with respect to the murine, but the predicted sequence does not. Schematically, being A, B, C possible aminoacids for the triplet (murine, humanized, predicted), we have: (A, A, A) → TN; (A, A, B), (A, B, C) → FP; (A, B, A) → FN; (A, B, B) → TP; thus, HD (p, h) = FP + FN. The True and False Positive Rates are defined as TPR = TP/(TP + FN); FPR = FP/(TN + FP). The last column is the Youden’s index: see Methods.

SAMC simulations start at high temperature (see Methods), which induces an immediate jump from the original murine score, with HD equal to zero, to very low scores and high distances from the initial murine sequence (see SI Fig. S4): the system explores sequences with basically all the residues mutated with respect to the original murine (since we fix 111 CDR positions, the maximal possible HD is 187). The system accepts changes with probability around 0.9, suggesting that the initial temperature is high enough to cross the “energy barriers” and freely explore the sequence space. During the simulation, the temperature is slowly reduced, up to a point where there is a sudden transition from an entropy-dominated regime, where the system explores many sequences with low score (high energy) to an energy-dominated region, where the system get stuck in a high score (low energy) region of the sequence space, ideally where the optimal sequence is located. Correspondingly, the Hamming distances suffer a sudden change as well, and reduce their fluctuations to a small region around their final value.

In Fig. 4 the course of the SAMC simulation can be followed by observing that all targets’ trajectories share an initial jump from HD = 0 to a region of high distance, close to the maximum possible one, and low MG score, and then move towards the final values of high MG-score and intermediate HD. Notice that the trajectories finally explore highly-human regions of several hundreds of sequences; however, there is no sampling of the region between the original murine sequence and the final basin of high-scoring sequences. On the other hand, the SD trajectory explores precisely this region, providing humanized sequences of increasing dissimilarity from the original sequence. Notably, the maximal score sequence obtained by SAMC usually does not coincide with (and, obviously, is better than) the best SD sequence: the difference can be of a few residues, pointing at essentially the same minimum in the sequence space, but also of more than 20 residues (such in the case of target 4), suggesting that in some case the SD evolution can become trapped in a local energy minimum, quite different from the global one. This could appear surprising, given that the energy function is a continuous function in *N* dimensions, with a unique global minimum; however, our dynamics is forced to be discrete, and we keep the CDR residues fixed: this could induce barriers in an otherwise funnel-like landscape, that can trap the SD trajectory, that is bound to find a local energy minimum, while the SAMC procedure has more chances to escape local traps and find the global optimum. On the other hand, the SD approach is convenient for our goals because it naturally provides, by construction, a trajectory of locally optimal solutions at any given distance from the original murine sequence, that is, the sequence of “best humanizing mutations” to perform, starting from the murine sequence; this is extremely useful within a conservative strategy to humanization, aiming at finding a functional humanized sequence rather than the best scoring one, while keeping the number of mutations as small as possible, in order to avoid affecting the stability or solubility of the original protein. The above results suggest that in a first approach, the SD trajectory could yield sufficient information to obtain reliable humanized sequences, at different similarity to the original murine one; SAMC could be unnecessary, unless the SD sequences prove to be unfit and there is a need to obtain an alternative list of several different high scoring sequences.

## Discussion

In this article we have introduced a statistical score, built on the Multivariate Gaussian Modeling^10^, that is more reliable, as a measure of the “degree of humanness”, than the distance of the query sequence from the ensemble of human sequences, or to a subset of it (the *T*_*n*_ methods). Indeed, we have seen that its performance in distinguishing human from murine sequences is higher than the methods relying on just the sequence similarity, due to the fact that the statistical model, on which the MG-score is based, accounts for pair-correlations between residues at different positions. Such correlations represent a key features for the performance of the model, that indeed drops dramatically if they are neglected. However, neglecting correlations between VH and VL regions does not affect the classification performance; since all our databases are restricted to VH-VL pairs that indeed are part of the same antibody, the irrelevance of correlations between VH and VL regions is not the artefact of a random juxtaposition of the two chains, but points to a very limited role of the interplay between the two regions, at least as far as classification is concerned. If translated to humanization tasks, this observation would support the common approach of humanizing the light and heavy chain independently.

The proposed humanness score shows a correlation with the experimental immunogenicity of therapeutic antibodies, even if such correlation is far from perfect. On the other hand, the comparison is made difficult by a series of factors affecting the experimental data: first, immunogenicity is studied in a clinical environment, measuring the frequency of anti-antibody reactions in sets of patients that necessarily represent inhomogeneous samples, due to their age, physical conditions etc.; on top of this, the same antibody, used for sets of patients affected by different diseases or in combination with other drugs, triggers different immunogenic responses. Also, even murine therapeutic antibodies, approved for long-duration treatment, are very likely to present little immunogenicity (imaging antibodies can have more, due to a more sporadic use), so that they are not expected to present the “typical immunogenicity” of a random murine antibody, and this surely introduces a bias in the data. Unfortunately, there is at present no alternative tool that can reliably predict the immunogenicity of a given antibody, so it was not possible to study the goodness of our humanness score as a immunogenicity predictor, for a sufficiently large dataset, free from the biases of therapeutic antibodies.

In any case, the analysis of murine-humanized antibody pairs reveals that, systematically, the MG-score increases upon humanization, thus indirectly confirming the value of the score for humanization tasks. Starting from the murine sequences, we have performed in-silico humanizations, that represents trajectories in the sequence space, leading to high-scoring sequences. The proposed SD/SAMC methods for optimizing the score produce a huge number of sequences beyond the threshold score, that could be considered as candidate humanized sequences. Most often, the SAMC procedure finds better solutions than the (much faster) SD, that might get trapped in suboptimal sequences. However, considering that in a humanization protocol it is more interesting to get good sequences with a minimal amount of mutations of the original sequence, rather than a global optimum, we have focused mainly in the SD trajectories originating at the different starting murine sequences. In all cases that we have analyzed, there are several common mutations between the last (and best) sequence in the SD procedure and the experimentally humanized sequence; the latter is not visited along the SD trajectory, even if this is not necessarily something to worry about, since there is no reason for the experimentally humanized sequence to be the only acceptable one. Interestingly, the optimal sequence that we find by SD is generally recognized by protein-BLAST as “more human” than the corresponding experimentally humanized one; this suggests that intermediate sequences found in the SD trajectory, with lower humanness score, could represent suitable candidates as well, and their higher similarity to the original functional sequence should reduce the risk of loosing stability or solubility.

So, within a humanization protocol, the advantage of our approach over CDR-grafting is that it proposes a precise set of candidate sequences, at increasing distance from the murine one and increasing humanness score, instead of requiring the introduction of arbitrary, manual back-mutations; such sequences can be shortlisted by further modelling of their structure and stability, while the final choice relies on the experimental result from the best hits. We stress that the method we propose represents an alternative approach to the usual grafting on germline sequences, since it deals with the statistical properties of the ensemble of mature sequences, and its performance can only improve as more sequences are collected, yielding better statistics. Indeed, the ensemble of rearranged human sequences that we use as learning set presents some of the desired properties for therapeutic antibodies, due to their nature (we expect that they share at least solubility and low immunogenicity); thus, as far as these properties can be recovered by a statistical model based on residue-residue interaction, we expect that an increase of the number of sequences in the database will yield high scoring sequences being indeed better humanized sequences.

Moreover, inferring a score from a database of experimentally verified sequences represents a flexible approach, that can be adapted to different learning databases. In perspective, this involves precision-medicine applications, resorting to individual antibody repertoires, that are becoming available through Rep-Seq techniques. Also, it can be easily adapted to veterinary drugs development, by changing the human learning dataset to the appropriate animal one. With the growing wealth of sequence databases, statistical-inference methods could become an increasingly relevant tool, with a range of applications that is still to be explored.

## Methods

We report here a general description of the methods: further details can be found in SI.

### Database preparation

#### Learning databases

We download from the IMGT/LIGM-DB server two databases with the whole set of human, rearranged, cDNA, VH sequences (11463 units) and VL sequences (5546 units), respectively, in the IMGT format. We extract and annotate each sequence with a unique identification strings that combines several fields of the IMGT record. To align the sequences, we resort to the ANARCI tool^18^ (version 1.1) with the AHo numbering scheme^19^, that is structurally motivated and basically free from insertions. We build a combined VH-VL database by matching VH and VL sequence according to their identification string, and joining them in a unique, aligned sequence of fixed length L = 298 (including gaps: the AHo scheme aligns both VH and VL in frameworks of 149 positions). All cases where the matching is not unique are removed. This yields a database of 1309 joint VH-VL sequences.

We perform the same steps on two databases with the whole set of mouse (“mus musculus”), rearranged, cDNA, VH (8389 units) and VL sequences (1514 units), both downloaded from the IMGT/LIGM-DB server, ending up with a combined database of 373 aligned sequences. Notice that we use the mouse learning database only for the classification with two reference distributions, while the rest of our results are based on just the human learning dataset.

#### Test databases

We download from the DIGIT server^20^ the whole database of matching human VH-VL sequences (3322 sequences), aligned according to the Kabat scheme. We remove the alignment, split the sequences into separate VH and VL chains, and filter on their length as above. We perform the same steps for the database of murine VH-VL matching sequences (1933 units.) Then, we use ANARCI to align the human VH and VL files according to the AHo scheme, and eliminate repeated sequences both within the DIGIT VH or VL files, as well as between these and the corresponding murine DIGIT aligned files, and the aligned human and mouse learning databases. Finally, we combine the VH and VL sequences, obtaining a database of 1388 sequences. The same procedure is repeated for the murine VH and VL files, yielding a combined database of 1379 sequences.

#### Humanized and Therapeutic antibodies database

We extract from DrugBank a list of therapeutic antibodies whose sequences are publicly available, reported in SI Table S1. For these sequences, we have also searched publicly available information on immunogenicity (as measured as the frequency of appearance of antidrug antibodies)^21–23^. The complete list of sequences, together with their reported immunogenicity, can be found in SI file “Therapeutic_Ab.txt”. Also, we compiled from literature^24–30^ a list of pairs of corresponding murine and experimentally humanized sequences, reported in SI Table S2.

#### Definition of the CDRs

We define the CDR regions according to the IMGT scheme: in the AHo layout, this imply the following definition: for the VH region (residues 1–149): 27–40 (CDR1), 58–68 (CDR2), 107–138 (CDR3); for the VL region (residues 150–298): 176–189 (CDR1), 207–217 (CDR2), 257–287 (CDR3). These positions are calculated with the following protocol: rather than resorting to the correspondence table reported in www.bioc.uzh.ch/plueckthun/antibody/Numbering/NumFrame.html we align some input sequences in the AHo scheme, and then query the IMGT-VQUEST^31^ server, to identify the CDR regions, and find their position in the alignment.

### Sequence Classifiers

We compare two different approaches, one based on the identity between pairs of sequences, and the latter based on the inference of a statistical model describing each (human or murine) sequence distribution.

#### Classifiers based on sequence identity

We define the distance between two sequences ${{\bf{A}}}^{1}=\{{A}_{i}^{1},i=\mathrm{1,}\,\ldots ,\,L\}$, ${{\bf{A}}}^{2}=\{{A}_{i}^{2},i=\mathrm{1,}\,\ldots ,\,L\}$, where *L* is the length of the alignment (including gaps) in the AHo numbering scheme, as: ${d}_{{{\bf{A}}}^{{\bf{1}}},{{\bf{A}}}^{{\bf{2}}}}={\sum }_{i\mathrm{=1}}^{L}\mathrm{(1}-{\delta }_{{A}_{i}^{1}{A}_{i}^{2}})$, where ${A}_{i}^{m}$ indicates the amino acid type at position *i* of sequence *m* and *δ*_*X*,*Y*_ is the Kronecker delta, equal to one or zero according to whether the residues *X* and *Y* are identical or not. From this, several different proximity scores can be defined, of the kind: ${T}_{k}({\bf{A}})=\frac{1}{k}{\sum }_{{\bf{a}}\in {{\mathscr{N}}}_{k}^{(h)}({\bf{A}})}d({\bf{A}},{\bf{a}})$ where ${{\mathscr{N}}}_{k}^{(h)}$ is the set of *k* sequences in the human learning dataset that are most similar to the query sequence **A**. Hence, *T*_1_(**A**) is the distance of **A** from the closest sequence in the human learning database, while we call *T*_*all*_ the average distance of **A** from the learning ensemble of human sequences: ${T}_{all}\equiv {\bar{d}}^{(h)}({\bf{A}})={T}_{{M}_{h}}({\bf{A}})$, with *M*_*h*_ the number of sequences in the human learning database. Analogously, it is possible to define the equivalent quantities (e.g. ${\bar{d}}^{(m)}({\bf{A}})$) referred to the the murine learning ensemble.

The classification with one reference distribution uses *T*_*k*_(**A**) as the score of **A**. A threshold score separating the human from non-human (mouse) class is defined for each method, by optimizing its performance on the test databases: each value of the threshold involves a different number of False Positives (*FP*) and True Positives (*TP*), which correspond to a point in the *FPR*-*TPR* plane, where the False Positive Rate (*FPR*) and True Positive Rate (*TPR*) are defined as: *TPR* = *TP*/*P*; *FPR* = *FP*/*N*, where *P* and *N* are, respectively, the number of human sequences (considered as Positive events), and of murine sequences (considered as Negatives) in the test databases. Varying the values of the threshold score, a curve (ROC curve) is drawn in the *FPR*-*TPR* plane, characterizing the goodness of the classifier: the bigger the area under the curve, the better is the classifier. The best threshold value can be determined as the one yielding the point on the curve that maximizes the Youden’s index (that in general, is also very close to the one maximizing the Matthews Correlation Coefficient): $Y=TP/(TP+FN)+TN/(TN+FN)-1$ or the Matthews Correlation Coefficient $M=\frac{(TP\cdot TN-FP\cdot FN)}{\sqrt{(TP+FP)(TP+FN)(TN+FP)(TN+FN)}}$. Notice that the threshold score identified in this way is maintained when analyzing the therapeutic antibodies. The classification with two reference distributions does not need the definition of a threshold score: it is based on the difference: $s({\bf{A}})={\bar{d}}^{(h)}({\bf{A}})-{\bar{d}}^{(m)}({\bf{A}})$, with positive values indicating that the sequence **A** is closer to the human ensemble (and therefore, human) while negative values correspond to murine sequences.

#### Classifiers based on inference of a probabilistic model

We follow Baldassi *et al*.^10^ and infer a multivariate Gaussian distribution from each VH, VL and combined VHVL learning databases, using uninformative prior distributions. From this, we calculate the posterior predictive distribution, that results to be a multivariate Student distribution, and use it to score the sequences in the test datasets. We sketch below the main tenets of the approach; more details can be found in SI Methods.

We start by mapping the L-residues-long, aligned sequences of the database (made up by M sequences, and drawn from a *Q* = 20 letters alphabet) to a binary sequence of *N* = *QL* bits $\{{x}_{i}=\mathrm{0,}\,\mathrm{1,}\,i=\mathrm{1,}\,\ldots ,\,N\}$, that, in block of *Q* bits, represent all the amino acids. As in ref.^10^ we assume that each of the *M* sequences in the database is drawn from a normal distribution with parameters *μ*, Σ (thus promoting ${x}_{i}^{m}$ to be real numbers):

1 $$p({x}^{m}|\mu ,{\rm{\Sigma }})={\mathscr{N}}(\mu ,{\rm{\Sigma }})$$

and we assume a Normal Inverse Wishart prior distribution for the parameters *μ*, Σ: $${p}^{pr}(\mu ,{\rm{\Sigma }})=$$ $${\mathscr{N}}\, {\mathcal I} \,{\mathscr{W}}(\eta ,{\rm{\kappa }},{\rm{\Lambda }},{\rm{\nu }})$$$$={\mathscr{N}}\,(\mu |\eta ,\frac{{\rm{\Sigma }}}{\kappa }) {\mathcal I} \,{\mathscr{W}}({\rm{\Sigma }}|{\rm{\Lambda }},{\rm{\nu }})$$. Using Bayes theorem, the posterior distribution for *μ*, Σ, given the data *X* can be calculated, yielding again a NIW distribution with new parameters $$\eta ^{\prime} $$, $${\rm{\kappa }}^{\prime} $$, Λ′, $${\rm{\nu }}^{\prime} $$:

2 $${p}^{post}(\mu ,{\rm{\Sigma }}|X)\propto p(X|\mu ,{\rm{\Sigma }}){p}^{pr}(\mu ,{\rm{\Sigma }})={\mathscr{N}}\, {\mathcal I} \,{\mathscr{W}}(\eta ^{\prime} ,{\rm{\kappa }}^{\prime} ,{\rm{\Lambda }}^{\prime} ,{\rm{\nu }}^{\prime} )$$

where the expression of $\eta ^{\prime} ,{\rm{\kappa }}^{\prime} ,{\rm{\Lambda }}^{\prime} ,{\rm{\nu }}^{\prime}$ can be found in Eq. (10) of ref.^10^ as a function of the empirical average $\bar{x}$ and empirical covariance ${\bar{C}}_{i,j}$ of the data. In a Bayesian approach, we derive the posterior predictive distribution for each new sequence $y=\{{y}_{i},i=\mathrm{1,}\,\ldots ,\,N\}$, by integrating on *μ*, Σ the joint probability: $p(y,\mu ,{\rm{\Sigma }}|X)=p(y|\mu ,{\rm{\Sigma }}){p}^{post}(\mu ,{\rm{\Sigma }}|X)$. Plugging Eqs (1) and (2) into the above equation, we get

3 $$p(y,\mu ,{\rm{\Sigma }}|X)=\rho (y)\,{\mathscr{N}}\, {\mathcal I} \,{\mathscr{W}}\,(\mu ,{\rm{\Sigma }}|\eta ^{\prime\prime} ,{\rm{\kappa }}^{\prime\prime} ,{\rm{\Lambda }}^{\prime\prime} ,{\rm{\nu }}^{\prime\prime} )$$

where the expressions for $\eta ^{\prime\prime} ,\,{\rm{\kappa }}^{\prime\prime} ,\,{\rm{\Lambda }}^{\prime\prime} ,\,{\rm{\nu }}^{\prime\prime}$ and ρ are reported in SI Methods. Upon integrating the above equation on *μ*, Σ, we get the posterior predictive distribution of a new sequence *y*, given the database of sequences *X*, as the multivariate t-distribution probability density:

4 $$p(y|X)={t}_{N}(\frac{M}{1-\lambda }+\mathrm{2,}{\langle \mu \rangle }_{post},(1+\frac{1-\lambda }{M}){\langle {\rm{\Sigma }}\rangle }_{post})$$

with: ${\langle \mu \rangle }_{post}=\lambda \eta +\mathrm{(1}-\lambda )\bar{x}$, ${\langle {\rm{\Sigma }}\rangle }_{post}=\lambda U+\mathrm{(1}-\lambda )\bar{C}+\lambda \mathrm{(1}-\lambda )\,(\bar{x}-\eta )\,{(\bar{x}-\eta )}^{T}$. We choose *η* and *U* as those corresponding to the mean and covariance estimates of a uniformly distributed sample.

We use the logarithm of *p*(*y*|*X*) in Eq. (4) as a score of the humanness of any given sequence *y*, and call it the “MG score”. Notice that *p*(*y*|*X*) is a probability density, and not a probability: as such, it is not bound between 0 and 1, and actually, due to its strong localization in the high dimensional sequence space, it will greatly exceed 1.

Finally, we have to choose a value for *λ* to be used in our inference, to set the best amount of regularization *λU* that should be added to the empirical covariance to optimize the statistical model. We do so by analyzing the different ROC curves, obtained at different values of *λ* in classifying the test databases (see the previous section for the definition of the ROC curve), and choosing the value of *λ* yielding the curve with the maximal area under it. Finally, for the classification with one reference distribution, we select, as the threshold score, the one corresponding to the point, on the ROC curve for the test database, with the highest value of the Youden’s coefficient. Notice that the threshold score identified in this way is maintained when analyzing the therapeutic antibodies, and in the humanization protocol.

When classifying with two distributions, we fix the *λ* for both the murine and human statistical models as explained above. Then, we simply score each query sequence in the test databases with both statistical models, classifying it as human or murine depending on the which of the two scores is higher.

Classification without correlations is performed by maintaining the same value of Ω and neglecting correlations between blocks of binary variables representing residues at different positions along the sequence; this ensures that Σ^−1^ will be block-diagonal as well, with no interactions between residues. We optimize *λ* as before, obtaining *λ* = 0.027. Analogously, classification without correlations between VH and VL regions is performed by maintaining the same value of Ω and asking that Σ*ij* = 0 if *k*, *l* belong to the VH and VL region, respectively. Again, the resulting Σ^−1^ will be block-diagonal, with no interactions between residues belonging to different variable regions. In this case, *λ* = 0.067.

### Humanization of murine sequences

We use the negative MG score, $E=-\,\mathrm{log}(p(y|X))$, see Eq. 4, as the objective function (the “Energy”, in analogy with statistical physics), that thus has to be minimized. We start from a murine sequence and, keeping fixed the positions corresponding to the CDR regions of the antibody, to ensure that it will continue recognizing the antigen, we mutate the other residues to make the sequence more human like. To this end, we perform two different strategies: 1) A steepest descent approach (SD), consisting in choosing, at each step, the mutation, at any position, that causes the biggest decrease in *E*. The process stops when all possible mutations at all sites increase the “energy”; 2) A Simulated Annealing Monte Carlo approach (SAMC), where we propose a random mutation at a random position, and accept it according to the Metropolis scheme. The simulated annealing starts at a high temperature, *T* = 25.1, and lowers the temperature in Δ*T* = 0.5, every $3.74\cdot {10}^{6}$ MC steps, until reaching *T* = 0.1. Here the temperature is just a parameter that controls the ability of the system to jump away from local minima and freely move through the sequence space (at high *T*), or to get trapped and explore the basins surrounding a minima, at low *T*.

The first technique finds the closest local minimum of the objective function in the neighborhood of the starting sequence, and the optimal path to reach it. Simulated Annealing, starting from high temperatures, explores wider regions of the sequence space, and in general finds the deepest minima, corresponding to higher humanness score.

We have tried both methods to humanize the 7 different murine sequences contained in the murine-humanized pairs dataset, SI Table S2. Since we know the corresponding experimentally humanized partner for each one, we can even control to what extent our theoretical humanized sequence matches its experimental counterpart.

## Electronic supplementary material

Supplementary Information

Datasets 1-5

## Data Availability

The learning and test datasets, that we have compiled from public sources and analysed during the current study, are available from the corresponding author on reasonable request.
