# Supplementary material for: Humanization of Antibodies using a Statistical Inference Approach
Source: Sci Rep. 2018 Oct 4;8:14820. doi: 10.1038/s41598-018-32986-y (PMC6172228; doi:10.1038/s41598-018-32986-y)
Supplement: Supplementary file 1 — Supplementary Information [file 41598_2018_32986_MOESM1_ESM.pdf]

Humanization of Antibodies using a Statistical Inference Approach – Supplementary Information

Alejandro Clavero-Álvarez[1], Tomas Di Mambro[2], Sergio Perez-Gaviro[3,4,1], Mauro Magnani[2], and Pierpaolo Bruscolini[1,4,*]

[1]Universidad de Zaragoza, Departamento de Física Teórica, Zaragoza, 50009, Spain
[2]Department of Biomolecular Sciences, University of Urbino ”Carlo Bo”, Urbino, Italy
[3]Centro Universitario de la Defensa, Zaragoza, 50090, Spain
[4]Universidad de Zaragoza, Instituto de Biocomputación y Física de Sistemas Complejos (BIFI), Zaragoza, 50018, Spain
[*]pier@unizar.es

ABSTRACT

Supplementary Results and Methods for the article.

Supplementary Methods

Database preparation

Learning databases

We download from the IMGT/LIGM-DB server two databases with the whole set of human, rearranged, cDNA, VH sequences (11463 units) and human, rearranged, cDNA, VL sequences (5546 units), respectively, in the IMGT format. We extract and annotate each sequence with: 1) its IMGT Id; 2) a unique string derived from the “partner” field (if present), or from the “publication title” and “sample” and “isolate” fields (if present); 3) the clone name (if present). For simplicity, the long string mentioned above at 2) is actually replaced by its encoded version using the Python module “hashlib.md5()”; the robustness of such encryption tool guarantees that different long strings will be transformed into different hash codes. The “clone name” information is not inserted in the larger string, since such field appears to be free text in the records, so that it must be dealt with separately. In this way, each sequence will be accompanied by an identification string as unique as possible, in order to recognize its partner sequence from the other database. Then, we analyse the length distribution of the sequences, (SI Fig S1), that reveals that they are clearly peaked; we decide to remove the outliers and keep just the sequences with length included in the range $[85,136]$ for VH, and $[79,118]$ for VL chains. We also remove sequences containing the “X” residue type, to keep just reliable amino acids.

To align the sequences, we resort to the ANARCI tool[1] (version 1.1) using the AHo numbering scheme[2], that we choose for being structurally motivated and basically free from insertions.

Once created the two separate databases, for the combined database we face the problem that often the VH and VL sequences are deposited separately, and only in a few cases there is the clear indication of the partnership between a VH and VL sequence in their records. To cope with it, we match any entry in our VH database with an entry from our VL database according to whether they have the same identification string (meaning that an explicit partnership information is provided in the record, or that they are published in the same article, possibly together with information on the “isolate” and “sample” they are extracted from). When the identification string is the same, but there are several sequences sharing the same string, we check the similarity between the name of the clones they correspond to, keeping just the cases where the names differ by just one “H” to “L” substitution (unfortunately, just using the publication yields a great number of false matches, since the same authors publish several sequences in the same paper, and the indication of the clone is optional and in free-text format). If the “clone” is not reported, and the identification string does not provide a unique match, the sequence is removed to avoid spurious matches.

The VH and VL sequences appearing in the curated combined database are then identified in the aligned databases, and joined according to their matching rules, to produce a final database of aligned constructs VH+VL. We end up with a database of 1309 combined VH and VL sequences.

We perform the same steps on two databases with the whole set of mouse (“mus musculus“), rearranged, cDNA, VH (8389 units) and VL sequences (1514 units), both downloaded from the IMGT/LIGM-DB server, ending up with a combined database of 373 aligned sequences.

For both human and mouse sequences, we also consider separately the VH and VL databases obtained by splitting the combined databases into their VH and VL parts (and removing repeated VH or VL chains, if necessary).

Notice that we use the mouse learning database only for the classification with two reference distributions, while the rest of our results are based on just the human learning dataset.

Test databases

We download from the DIGIT server[3] the whole database of matching human VH/VL sequences (3322 sequences), aligned according to the Kabat scheme. We remove the alignment, split the sequences into separate VH and VL chains, and filter on their length as above. We perform the same steps for the database of murine VH/VL matching sequences (1933 units.)

Then, we use ANARCI to align the human VH and VL files according to the AHo scheme, and eliminate repeated sequences both within the DIGIT VH or VL files, as well as between these and the corresponding murine DIGIT aligned files, and the aligned human and mouse learning databases. Finally, we combine the VH and VL sequences, obtaining a database of 1388 sequences.

Analogously, we use ANARCI to align the murine VH and VL files according to the AHo scheme, eliminating repeated sequences both within the DIGIT VH or VL files, and between these and the corresponding human DIGIT aligned files, and the aligned human and mouse learning databases. Finally, we combine the VH and VL sequences in a database of 1379 sequences.

Again, for both human and mouse sequences, we also consider the VH and VL databases obtained by splitting the combined databases.

Humanized and Therapeutic antibodies database

Table S1. List of therapeutic antibodies.

	-umab	-zumab	-ximab	-omab
1	Adalimumab	Alemtuzumab	Abciximab	3F8
2	Denosumab	Atezolizumab	Basiliximab	81C6
3	Durvalumab	Bevacizumab	Bavituximab	Arcitumomab
4	Ipilimumab	Certolizumab pegol	Cetuximab	Blinatumomab chain 1
5	Necitumumab	Daclizumab	Rituximab	Blinatumomab chain 2
6	Nivolumab	Elotuzumab	Siltuximab	Ibritumomab
7	Ofatumumab	Farletuzumab		Muromonab
8	Olaratumab	Gemtuzumab chain 2		Satumomab
9	Ramucirumab	Idarucizumab		Tositumomab
10	Secukinumab	Ixekizumab		
11	Zalutumumab	Matuzumab chain 1		
12		Matuzumab chain 2		
13		Omalizumab		
14		Oportuzumab		
15		Pembrolizumab		
16		Pertuzumab		
17		Pexelizumab		
18		Ranibizumab		
19		Trastuzumab		
20		Vedolizumab		

	VH	VL	Ref.
Seq. 1 M	QVQLQQSGPEVVRPGVSVKISCKGSGYIFTDYA MHWVKQSHAKSLEWIGVISTYTGKTNYSQKFKG KATMTVDKSSSTAYMELARLTSEDSSVYYCARR GDYDAWFAYWGQGTLVTVSA	DIVMSQSPSSLAVSVGEKVSMTCKSSQRLLYSS DQKNYLAWYQQKPGQSPKVLIYWASTRVSGVPD RFTGSESGTDFTLTISSVKAEDLAVYYCQQYYT YPLTFGAGTKLELK	[4]
Seq. 1 H	QVQLVQSGAEVKKPGASVKVSCKASGYTFTDYA MHWVRQAPGQGLEWIGVISTYTGKTNYSQKFKG RATMTVDKSISTAYMELSRLRSDDTAVYYCARR GDYDAWFAYWGQGTLVTVSS	DIVMTQSPDSLAVSLGERATMNCKSSQRLLYSS DQKNYLAWYQQKPGQPPKVLIYWASTRVSGVPD RFSGSESGTDFTLTISSLQAEDVAVYYCQQYYT YPLTFGQGTKVEIK	[4]

Seq. 2 M	QVQLQESGPELVKPGASMKISCKASGYSFTEHI<br>INWVKQTHRENLEWIGLINPNSGGTNYNQKFKD<br>KATLTVDTASNTAYMELLSLTSEDSAVYYCARL<br>RYDAAYWGQGTTVTVSS	DIELTQSHKFMSTSVGDRVSITCKASQDVTAAV<br>AWYQQKPGQSPKLLIHSASYRYTGVPDRFTGSG<br>SGSDFTFTISSVQAEDLAVYYCQQYYNTPLTFG<br>AGTKLELK	[5]
Seq. 2 H	QVQLVQSGAEVKKPGASVKVSCKASGYTFTEHI<br>INWVRQAPGQNLEWMGLINPNSGGTNYNQKFKD<br>RVTMTTDTSTSTAYMELLSLRSDDTAVYYCARL<br>RYDAAYWGQGTTVTCSS	DIQMTQSPSSLSASVGDRVTITCKASQDVTAAV<br>AWYQQKPGKAPKLLIYSASYRYTGVPSRFSGSG<br>SGTDFTLTYSSLQPEDFATYYCQQYYNTPLTFG<br>GGTKVEIK	[5]
Seq. 3 M	QVTLKESGPGILQPSQTLSLTCSFSGFSLRTSG<br>MGVGWIRQPSGKGLEWLAHIWWDDDKRYNPALK<br>SRLTISKDTSSNQVFLKIASVDTADTATYYCAQ<br>INPAWFAYWGQGTLVTVS	DIVLTQSPASLAVSLGQRATISCKASQSVDFDG<br>DSFMNWYQQKPGQPPKLLIYTTSNLESGIPARF<br>SASGSGTDFTLNIHPVEEEDTATYYCQQSNEDP<br>YTFGGGTKLELK	[6]
Seq. 3 H	QVTLKESGPALVKPTQTLTLTCTFSGFSLSTSG<br>MGVGWIRQPPGKALEWLAHIWWDDDKRYNPALK<br>SRLTISKDTSKNQVVLTMTNMDPVDTATYYCAR<br>INPAWFAYWGQGTLVTVSS	DIVMTQSPDSLAVSLGERATINCKASQSVDFDG<br>DSFMNWYQQKPGQPPKLLIYTTSNLESGVPDRF<br>SGSGSGTDFTLTIRPLQAEDVAVYYCQQSNEDP<br>YTFGQGTKLEIK	[6]
Seq. 4 M	EVQLEESGTELARPGASVKLSCKASGYIFSGYW<br>MQWIKQRPGQGLEWIGAIYPGDGDTRYTQKFKG<br>KATLTADKSSSTAYMQLSSLASEDSAVYYCAGG<br>NFFFDYWGLGTTLAVSS	DIVITQSPALMAASPGEKVTITCSVSSSISSSY<br>LHWYQQKSGISPKPWIYGTSNLASGVPARFSGS<br>GSGTSYSLTITSMEAEDAATYYCQQWSSSPLTF<br>GAGTKLELK	[7]
Seq. 4 H	QVQLVQSGAEVKKPGSSVKVSCKASGYTFSGYW<br>MQWIKQAPGQGLEWIGAIYPGDGDTRYTQKFKG<br>RATLTADKSTSTAYMELSSLRSEDTAVYYCAGG<br>NFFFDYWGQGTLVTVSS	EIVITQSPDFQSVTPKEKVTITCSVSSSISSSY<br>LHWYQQKPDQSPKPWIYGTSNLASGVPSRFSGS<br>GSGTDYTLTINSLEAEDAATYYCQQWSSSPLTF<br>GQGTKLEIK	[7]
Seq. 5 M	EVQILETGGGLVKPGGSLRLSCATSGFNFNDYF<br>MNWVRQAPGKGLEWLAQMRNKNYQYGTYYAESL<br>EGRVTVSRDDAKNSVYLQVSSLRAEDTAIYYCT<br>RESYYGFTSYWGQGVMVTVSS	DIQMTQSPASLSASLEEIVTITCQASQDIGISL<br>SWYQQKPGRTPQLLIQNANNLADGVPSRFSGRR<br>FGTQFSLTISTPQVEDTGVYYCLQHNSAPYTFG<br>TGTQLEIK	[8]
Seq. 5 H	EVQLVESGGGLVQPGGSLRLSCAASGFNFNDYF<br>MNWVRQAPGKGLEWVAQMRNKNYQYGTYYAESL<br>EGRFTISRDDSKNSLYLQMNSLKTEDTAVYYCA<br>RESYYGFTSYWGQGTLVTVSS	DIQMTQSPASLSASVGDRVTITCQASQDIGISL<br>SWYQQKPGKAPKLLIYNANNLADGVPSRFSGSG<br>SGTDFTLTISSLQPEDFATYYCLQHNSAPYTFG<br>QGTKLEIK	[8]
Seq. 6 M	GVQLQQSGPELVKTGASVKISCKASGYSFTDYF<br>MHWVKQSHGKSLDWIGYINCYTGATNYSQKFKG<br>KATFTVDTSSNTAYMQFNSLTSEDSAVYYCART<br>SIGYGSSPPFPYWGQGTLVTVSS	ETTVTQSPASLSMSIGEKVTIRCITSTDIDDDM<br>NWYQQKPGEPPRLLISDGNTLRPGVPSRFSSSG<br>YGTDFVFTIENMLSEDVADYYCLQSDNLPTYTF<br>GGGTNLEIK	[9]
Seq. 6 H	EVQLVQSGAEVKKPGATVKISCKVSGYTFTDYF<br>MHWVQQAPGKGLEWMGYINCYTGATNYSQKFKG<br>RVTITADTSTDTAYMELSSLRSEDTAVYYCATT<br>SIGYGSSPPFPYWGQGTLVTVSS	ETTLTQSPASLSMSIGEKVTIRCITSTDIDDDM<br>NWYQQKPGEPPRLLISDGNTLRPGVPSRFSSSG<br>YGTDFVFTIENMLSEDVAYYYCLQSDNLPTYTF<br>GGGTNLEIK	[9]
Seq. 7 M	QVQLQQSGAELVEPGASVKLSCKASGYNFANFF<br>IYWVQQRPGQGLEWIGEINPKHGDVKIHEKFKT<br>KATLTVDTSSNTAYIQLSSLTSEDSAVFYCTRG<br>DKFDGFDYWGQGTTLTVSS	HIVLTQSPAILSASPGEKVTMTCSANSGVNFMH<br>WYQQKSGASPKRYIYDTTELASGVPARFSASGS<br>GTSYSLTISSMEAEDSATYFCQQWSSNLPTFGS<br>GTKLEIK	[10]
Seq. 7 H	QVQLVQSGAEVKKPGASVKVSCKASGYNFANFF<br>IYWVRQAPGQGLEWIGEINPKHGDVKIHEKFKT<br>RATLTVDTSTSTAYMELSSLRSEDTAVYYCARG<br>DKFDGFDYWGQGTLVTVSS	DIVLTQSPAFLSVTPGEKVTITCSANSGVNFMH<br>WYQQKPDQAPKRYIYDTTELASGVPSRFSASGS<br>GTDYTFTISSLEAEDAATYFCQQWSSNLPTFGQ<br>GTKVEIK	[10]

Table S2. **Murine (M) and experimentally humanized (H) sequence pairs.**

Sequence Classifiers

Classifiers based on inference of a probabilistic model

We follow Baldassi et al.[11] and infer a multivariate gaussian distribution from each VH, VL and combined VHVL learning databases, using uninformative prior distributions. Then, we calculate the posterior predictive distribution, that results to be a multivariate Student distribution, and use it to score the sequences in the test datasets.

We start by mapping the L-residues-long, aligned sequences of the database (made up by M sequences, and drawn from a $Q = 20$ letters alphabet) to a binary sequence of $N = QL$ bits $\{x_i = 0, 1, i = 1, \ldots, N\}$, that, in block of Q bits, represent

all the amino acids. Namely, residue of type $a = 1,\dots,Q$ at position $k = 1,\dots,L$ of a given sequence, is mapped to a binary variable $x_{i=(k-1)Q+a}$. In principle, in any block of Q bits only one bit (or none, to represent a gap) can be set to 1, to prevent the coexistence of more than one amino acid at a site. In practice, following Baldassi et al.[11], we do not impose such constraint, letting the system itself implementing it through anticorrelations; we also introduce weights w_m for each sequence in the alignment, in order to calculate the empirical average and covariance:

$$\bar{x}_i = \frac{1}{M_e} \sum_{m=1}^{M} x_i^m w_m \;\; (i = 1,\dots,L) \tag{1}$$

$$\bar{C}_{ij} = \frac{1}{M_e} \sum_{m=1}^{M} (x_i^m - \bar{x}_i)(x_j^m - \bar{x}_j) w_m \tag{2}$$

with $M_e = \sum_{m=1}^{M} w_m$. The weights are calculated as $w_m = 1/n_m$, where:

$$n_m = \sum_{l=1}^{M} \theta(\mathscr{S}_{lm} - L\Omega) \tag{3}$$

is the number of sequences whose similarity $\mathscr{S}_{lm}$ with sequence m exceeds a threshold $L\Omega$. Here $\theta(\bullet)$ is the step function; the similarity is defined in terms of the number of identical residues (excluding gaps): $\mathscr{S}_{lm} = \sum_{i=1}^{L} \delta_{A_i^l A_i^m}(1 - \delta_{A_i^l,'-'})$, where A_i^mindicates the amino acid type at position i of sequence m and $\delta_{X,Y}$ is the Kronecker delta. At difference from the choice in[11], the threshold Ω is defined as the value that maximizes the Frobenius norm of the resulting $\bar{C}_{ij}$.

As in Ref.[11], we assume that each of the M sequences in the database, $x^m = \{x_i^m, i = 1,\dots,N\}$, is drawn from a normal distribution with parameters μ, Σ (thus promoting x_i^m to be real numbers):

$$p(x^m|\mu,\Sigma) = \mathscr{N}(\mu,\Sigma) = (2\pi)^{-\frac{N}{2}} |\Sigma|^{-\frac{1}{2}} \exp\left(-\frac{1}{2}(x^m - \mu)^T \Sigma^{-1} (x^m - \mu)\right) \tag{4}$$

with $|\Sigma|$ indicating the determinant of the $N \times N$ matrix Σ. In order to infer the best values of μ, Σ from the known database of human sequences $X = \{x^m, m = 1\dots,M\}$, we also assume a Normal Inverse Wishart prior distribution for the parameters μ, Σ:

$$\begin{aligned} p^{pr}(\mu,\Sigma) &= \mathscr{N}\mathscr{I}\mathscr{W}(\eta,\kappa,\Lambda,\nu) = \mathscr{N}(\mu|\eta,\frac{\Sigma}{\kappa})\mathscr{I}\mathscr{W}(\Sigma|\Lambda,\nu) = \\ &= \frac{2^{-\frac{N}{2}(1+\nu)} \pi^{-\frac{N}{4}(1+N)} \kappa^{\frac{N}{2}} |\Lambda|^{\frac{\nu}{2}}}{|\Sigma|^{\frac{1}{2}(2+N+\nu)} \prod_{n=1}^{N} \Gamma(\frac{\nu+1-n}{2})} \exp\left(-\frac{1}{2}\sum_{i,j=1}^{N} [\kappa((\mu_i - \eta_i)(\mu_j - \eta_j) + \Lambda_{ij})(\Sigma^{-1})_{ji}]\right) \end{aligned} \tag{5}$$

Using Bayes theorem, the posterior distribution for μ, Σ, given the data X can be calculated, yielding again a NIW distribution with new parameters η', κ', Λ', ν':

$$p^{post}(\mu,\Sigma|X) \propto p(X|\mu,\Sigma) p^{pr}(\mu,\Sigma) = \mathscr{N}\mathscr{I}\mathscr{W}(\eta',\kappa',\Lambda',\nu') \tag{6}$$

where

$$\begin{aligned} \kappa' &= \kappa + M = \frac{M}{1-\lambda} \\ \eta' &= \frac{\kappa}{\kappa+M}\eta + \frac{M}{\kappa+M}\bar{x} = \lambda\eta + (1-\lambda)\bar{x} \\ \nu' &= \nu + M \\ \Lambda' &= \Lambda + M\bar{C} + \frac{\kappa M}{\kappa+M}(\bar{x}-\eta)(\bar{x}-\eta)^T = \Lambda + M\bar{C} + \lambda M(\bar{x}-\eta)(\bar{x}-\eta)^T \end{aligned} \tag{7}$$

with

$$\lambda = \kappa/(\kappa+M) \tag{8}$$

The mean of μ and Σ with the posterior NIW distribution are given by

$$\langle\mu\rangle_{post} = \eta' \qquad \langle\Sigma\rangle_{post} = \Lambda'/(\nu' - N - 1) \tag{9}$$

while the mode of the distribution is achieved for $\mu = \eta'$, $\Sigma = \Lambda'/(\nu' - N + 1)$. In a frequentist approach, we could fix μ, Σ to their modes, and use Eq.(4) to draw new (humanized) sequences from the distribution. However in a Bayesian approach, we recognize that we do not know the "correct value", but just the distribution of μ, Σ, so we derive the posterior predictive distribution for each new sequence $y = \{y_i, i = 1, \ldots, N\}$, by integrating on μ, Σ the joint probability: $p(y, \mu, \Sigma|X) = p(y|\mu, \Sigma)\, p^{post}(\mu, \Sigma|X)$. Plugging Eqs. (4), (6) into the above equation, we get

$$p(y, \mu, \Sigma|X) = \rho(y)\, \mathscr{N}\mathscr{I}\mathscr{W}(\mu, \Sigma|\eta'', \kappa'', \Lambda'', \nu'') \tag{10}$$

where $\prod'' = \prod_{n=1}^{N} \Gamma(\frac{\nu''+1-n}{2})$,

$$\begin{aligned}
\kappa'' &= \kappa' + 1 \\
\eta'' &= \frac{\kappa'}{\kappa'+1}\eta' + \frac{1}{\kappa'+1} y \\
\nu'' &= \nu' + 1 \\
\Lambda'' &= \Lambda''(y) = \Lambda' + \frac{\kappa'}{\kappa'+1}(y - \eta')(y - \eta')^T \\
\rho(y) &= \pi^{-\frac{N}{2}} \left(\frac{\kappa'}{\kappa'+1}\right)^{\frac{N}{2}} \frac{|\Lambda''(y)|^{-\frac{\nu'+1}{2}}}{|\Lambda'|^{-\frac{\nu'}{2}}} \frac{\prod''}{\prod'}
\end{aligned} \tag{11}$$

with $\prod' = \prod_{n=1}^{N} \Gamma(\frac{\nu'+1-n}{2})$. Due to the normalization of the NIW distribution to 1, upon integrating Eq. (10) on μ, Σ, and using the Sylvester determinant identity $|\mathbb{I}_m + AB| = |\mathbb{I}_n + BA|$ (where A is a $m \times n$ and B an $n \times m$ matrix), we get the posterior predictive distribution of a new sequence y given the database of sequences X:

$$p(y|X) = t_N(\nu' - N + 1, \langle\mu\rangle_{post}, \frac{\kappa'+1}{\kappa'} \langle\Sigma\rangle_{post}) \tag{12}$$

where we have introduced the multivariate t-distribution probability density:

$$t_p(\rho, \mu, S) = \frac{\Gamma(\frac{\rho+p}{2})}{\Gamma(\frac{\rho}{2})(\rho\pi)^{\frac{p}{2}}} |S|^{-\frac{1}{2}} \left(1 + \frac{1}{\rho}(y-\mu)^T S^{-1}(y-\mu)\right)^{-\frac{\rho+p}{2}} \tag{13}$$

and we have used Eq. 9 to eliminate η', Λ'.

Finally, using Eqs. (7), (8) and choosing, as in Ref.[11], $\nu = N + \kappa + 1$, $\langle\Sigma\rangle_{prior} \equiv \frac{\Lambda}{\nu - N - 1} = U$, and the prior estimates η and U as those corresponding to the mean and covariance estimates of a uniformly distributed sample, we get:

$$p(y|X) = t_N\left(\frac{M}{1-\lambda} + 2, \langle\mu\rangle_{post}, \left(1 + \frac{1-\lambda}{M}\right)\langle\Sigma\rangle_{post}\right) \tag{14}$$

with:

$$\begin{aligned}
\langle\mu\rangle_{post} &= \eta' = \frac{\kappa}{\kappa+M}\eta + \frac{M}{\kappa+M}\bar{x} = \lambda\eta + (1-\lambda)\bar{x} \\
\langle\Sigma\rangle_{post} &= \frac{\Lambda'}{\nu' - N - 1} = \lambda U + (1-\lambda)\bar{C} + \lambda(1-\lambda)(\bar{x}-\eta)(\bar{x}-\eta)^T
\end{aligned} \tag{15}$$

We use the logarithm of $p(y|X)$ in Eq. (14) as a score of the humanness of any given sequence y, and call it the "MG score". Notice that $p(y|X)$ is a probability density, and not a probability: as such, it is not bound between 0 and 1, and actually, due to its strong localization in the high dimensional sequence space, it will greatly exceed 1.

Finally, we have to choose a value for λ to be used in our inference, to set the best amount of regularization λU that should be added to the empirical covariance to optimize the statistical model.

We do so by analyzing the different ROC curves, obtained at different values of λ in classifying the test databases (see the previous section for the definition of the ROC curve), and choosing the value of λ yielding the curve with the maximal area under it. Finally, for the classification with one reference distribution, we select, as the threshold score, the one corresponding to the point, on the ROC curve for the test database, with the highest value of the Youden's coefficient. Notice that the threshold score identified in this way is maintained when analyzing the therapeutic antibodies, and in the humanization protocol.

When classifying with two distributions, we fix the λ for both the murine and human statistical models as explained above. Then, we simply score each query sequence in the test databases with both statistical models, classifying it as human or murine depending on the which of the two scores is higher.

Correlation-neglecting classification Classification without correlations is performed by maintaining the same value of Ω and asking that $\Sigma_{ij} = 0$ if $k \neq l$, where $i = (k-1)Q + a$, $j = (l-1)Q + b$, $k,l = 1,\dots,L$, $a,b = 1,\dots,Q$, i.e. neglecting correlations between blocks of binary variables representing residues at different positions along the sequence; this ensures that Σ^{-1} will be block-diagonal as well, with no interactions between residues. This also yields that we can keep only the terms corresponding to the block diagonal terms in the matrices multiplying Σ^{-1}, due to the fact that the trace in the argument of the exponential in, for instance, Eq. (10) will be the same. Thus, a part from a different normalization in front of Eq. 14, the expressions Eqs. (14), (15) will still be valid, with block-diagonal matrices. We optimize λ as before, obtaining $\lambda = 0.027$.

Analogously, classification without correlations between VH and VL regions is performed by maintaining the same value of Ω and asking that $\Sigma_{ij} = 0$ if k,l belong to the VH and VL region, respectively, i.e., $i = (k-1)Q + a$, $j = (l-1)Q + b$, $k,l = 1,\dots,L/2$, $a,b = L/2+1,\dots,Q$ (since in AHo numbering scheme, the alignments of VH and VL regions have the same length). Again, the resulting Σ^{-1} will be block-diagonal, with no interactions between residues belonging to different variable regions. The value of λ in this case is $\lambda = 0.067$.

Definition of an Immunogenic Score

We run the MHCII program (IEDB_MHC_II, version 2.16.2: http://tools.iedb.org/mhcii/download/)[12,13] mhc_II_binding.py with the ”IEDB_recommended“ flag. Such program provides in output a list of 15-residues-long peptides, with several scores associated to them: we decide to use the percentile score as a reference, as it is a consensus score of different methods. According to the instructions, low percentile scores correspond to peptides that are more easily bound and recognized by T-cells, eliciting immunogenic response. However, there is not a clear rule to state when an antibody sequence will cause such response. We tried several quantities to define an immunogenic score: average percentile scores of the k lowest scoring peptides, number of alleles recognizing a peptide with percentile score below a certain threshold x_0, number of peptides within the antibody with a percentile score below a certain threshold x_0; we finally decide for the latter, which is the one that best distinguish human from murine sequences of the learning databases, according to the ROC curves reported in Table S2. Given a threshold number s of peptides with percentile score less than x_0, we define as “immunogenic” an antibody that presents more than s peptides below the percentile threshold x_0, and will consider as True Positives the murine sequences that are “immunogenic”, False Positives the human sequences that are “immunogenic”, and so on. Such definitions allow to calculate False Positive Rates and True Positive Rates for each value of s: upon varying “s”, we obtain a ROC curve, for any given value of the percentile threshold x_0.

Notably, the value $x_0 = 0.31$ corresponds both to the highest AUC (0.698203), and to the highest maximal values of Matthews Correlation Coefficient (MCC=0.294284, obtained for $s = 23$), while Youden’s J Statistic takes its maximal value at $x_0 = 0.30$ ($s = 15$, YJS=0.305817). We decide to take $x_0 = 0.31$ as the working value that best allows to distinguish human from murine. So, it will be considered as immunogenic as a mouse sequence, a VHVL sequence presenting globally $p \geq 23$ short peptides (of length 15) with a consensus percentile score $x \leq 0.31$.

Supplementary Results

Figure S1. Distribution of the sequence lengths for VH (Left) and VL (Right) sequences.

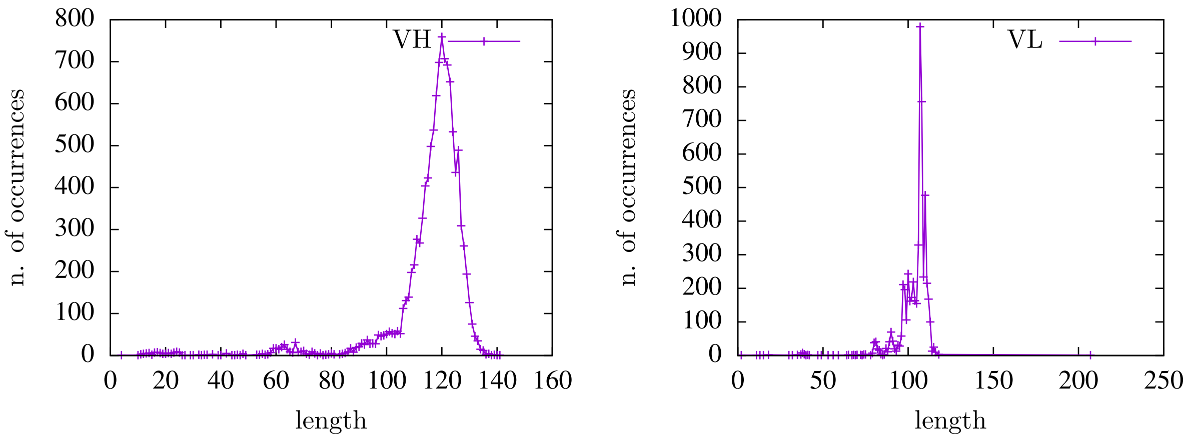

Table S3. **Quality indicators for the classification of the test database with different methods.** Values of the Area Under the Curve (AUC), the Matthews Correlation Coefficient (MCC), and Youden's index (YJS) obtained with different methods (MG, T_{all}, T_{20} and T_1) for classificatios based on the VH, VL, or both regions. For MCC and YJS, the table reports pairs (s, b) where s is the value of the score (either MG, T_{all}, T_{20} or T_1) yielding the maximal value of the MCC or YJS, and b is the maximal value itself. We report T_{20} in the comparison as it is the value proposed in Ref.[14].

	**VH region**			
	MG	T_{all}	T_{20}	T_1
AUC	0.940	0.710	0.908	0.923
max MCC (score, value)	(0.708, 2579)	(212, 0.405)	(118, 0.694)	(183, 0.718)
max YJS (score, value)	(0.772, 2598)	(213, 0.400)	(118, 0.694)	(183, 0.717)
	**VL region**			
	MG	T_{all}	T_{20}	T_1
AUC	0.980	0.784	0.754	0.720
max MCC (score, value)	(0.872, 3553)	(246, 0.460)	(236, 0.400)	(235, 0.355)
max YJS (score, value)	(0.925, 3628)	(246, 0.455)	(236, 0.400)	(235, 0.346)
	**VH - VL regions**			
	MG	T_{all}	T_{20}	T_1
AUC	0.969	0.815	0.944	0.949
max MCC (score, value)	(0.892, 6384)	(114, 0.552)	(74, 0.787)	(65, 0.824)
max YJS (score, value)	(0.891, 6383)	(116, 0.539)	(75, 0.785)	(65, 0.822)

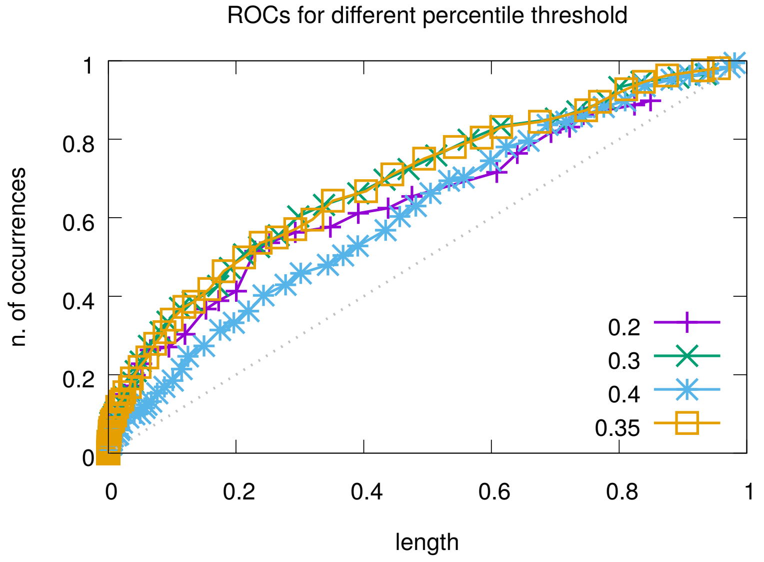

Figure S2. **ROC curves for the immunogenic score, calculated on the human and murine learning databases.** The ROC curves are calculated for different values of the percentile score below which peptides are considered immunogenic. Each curve is obtained by reporting TPR vs FPR at varying value of the the threshold number of peptides, per sequence, that are found below the threshold. That is, at a given value of the percentile score x_0, we assume that a sequence containing more than n_0 peptides with MHCII percentile score less than x_0 will be immunogenic, and calculate the FPR and TPR values at varying n_0: we consider a murine antibody with more than n_0 peptides with a percentile score below x_0, as a True Positive; a True Negative will be a human antibody with less than n_0 peptides below n_0. We observe that the immunogenic score is not very efficient in distinguishing human from murine sequences: the area under the ROC curve is just $AUC = 0.698$ in the best case.

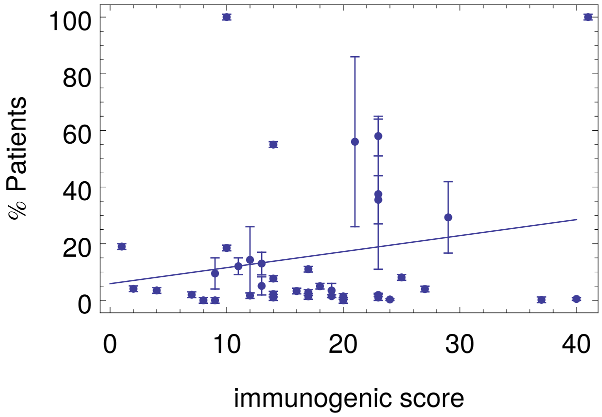

Figure S3. **Scatter plot of the experimental immunogenicity vs the immunogenic score.** The agreement is worse than that with the MG score, reported in the main text: now the linear fit is $y = 5.8922 + 0.565346x$, $R^2 = 0.042$ and a correlation coefficient of $C = 0.20$.

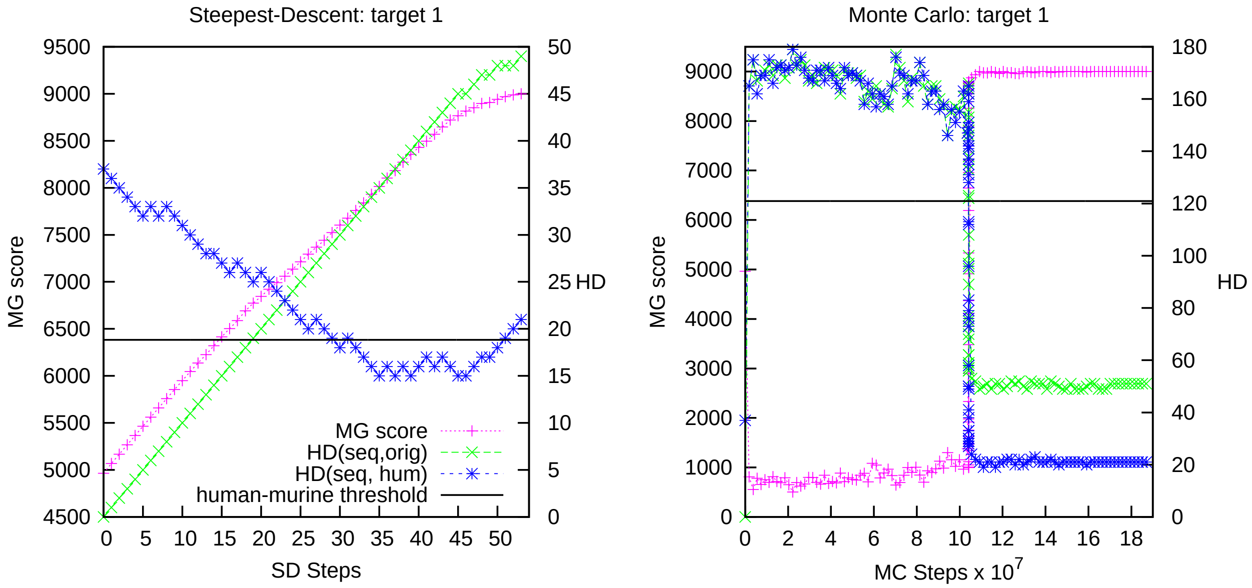

Figure S4. MG-score (left y-axis) and Hamming distance (right y-axis) for both the Steepest Descent (left) and the Simulated Annealing Monte Carlo (right) for our first murine target. The Hamming distance of the sequence at each step ("seq") to both the original ("orig"), murine sequence and the experimentally humanized one ("hum") are reported; color codes are the same in both panels.

Table S4. BLAST results on simulated and experimentally humanized sequences. We run protein-BLAST[15] separately on the VH and VL regions of the optimal SD sequences and the experimentally humanized ones, and report the most similar sequence found by BLAST: "Seq" indicates the target sequence, "MG" is the MG-score (in parentheses, the MG-score of the original murine sequence), and the rest of the columns report the output from protein-BLAST, run with the standard parameters, about the most similar sequence to the query one: the e-value is the measure of the quality of the identification (the lower, the better), the % identity refers to the sequence homology between the query and the best retrieved sequence, identified by the description and the accession number. The target sequence is identified as in the following examples: 1 EH H: H chain of the Experimentally Humanized (EH) sequence of target 1; 1 SD L: L chain of our SD Humanized sequence of target 1.

Seq.	MG	Description	e-val	% Id.	Accession
1 EH H	7571 (4963)	immunoglobulin gamma chain variable region [Mus musculus]	4e-65	83	AOO95240.1
1 EH L		immunoglobulin kappa light chain variable region [Homo sapiens]	3e-70	91	ACJ71709.1
1 SD H	9000 (4963)	immunoglobulin heavy chain variable region [Homo sapiens]	6e-65	86	AAA17912.1
1 SD L		immunoglobulin variable kappa chain [Homo sapiens]	4e-73	95	CAJ57195.1
2 EH H	7184 (4905)	anti-Sm antibody VH chain (VH1/DK1 or DM1/JH4b) - human	2e-61	82	S49530
2 EH L		Chain B, Structure Of Myostatin In Complex With Humanized Rk35 Antibody	6e-66	93	5F3H_B
2 SD H	9328 (4905)	anti-Sm antibody VH chain (VH1/DK1 or DM1/JH4b) - human	9e-72	91	S49530
2 SD L		immunoglobulin kappa light chain variable region [Homo sapiens]	5e-66	93	AAZ09096.1
3 EH H	6780 (4976)	anti-Sm antibody VH chain (VH1/DK1 or DM1/JH4b) - human	2e-61	82	S49530
3 EH L		monoclonal antibody kappa light chain variable region [Mus musculus]	2e-65	86	AAQ24153.1
3 SD H	7817 (4976)	immunoglobulin heavy chain variable region [Homo sapiens]	7e-72	91	ABK81412.1
3 SD L		Chain L, Cryst Structure Of Antigen-Binding Fragment From A Humanized Version Of The Anti-Human Fas Antib Hfe7a	2e-64	86	1IT9_L
4 EH H	5689 (4734)	immunoglobulin mu chain [Mus musculus]	2e-62	80	AAA88256.1
4 EH L		immunoglobulin light chain variable region [Homo sapiens]	1e-52	81	AKU38944.1
4 SD H	9362 (4734)	immunoglobulin heavy chain VHDJ region [Homo sapiens]	3e-69	89	BAC01347.1
4 SD L		anti-rabies virus immunoglobulin light chain variable region [Homo sapiens]	2e-63	94	AAY33350.1
5 EH H	7202 (4978)	Chain H, Crystal Structure Of The Fab Portion Of Olokizumab In Complex With Il-6	1e-83	100	4CNI_H
5 EH L		Chain L, Crystal Structure Of The Fab Portion Of Olokizumab In Complex With Il-6	6e-72	99	4CNI_L
5 SD H	8131 (4978)	Chain H, Crystal Structure Of The Fab Portion Of Olokizumab In Complex With Il-6	5e-75	91	4CNI_H
5 SD L		Chain L, Crystal Structure Of The Fab Portion Of Olokizumab In Complex With Il-6	2e-67	94	4CNI_L
6 EH H	5193 (4350)	immunoglobulin heavy chain variable region [Mus musculus]	2e-65	78	ABP04229.1
6 EH L		immunoglobulin light chain variable region [Mus musculus]	2e-69	97	ABP04230.1
6 SD H	8159 (4350)	circulating B cell antibody heavy chain variable region [Homo sapiens]	5e-68	87	ABF83408.1
6 SD L		immunoglobulin kappa light chain [Homo sapiens]	9e-62	89	BAH04696.1
7 EH H	5468 (4481)	Chain H, Structure Of Cd40l In Complex With The Fab Fragment Of Humanized 5c8 Antibody	2e-60	77	1I9R_H
7 EH L		antigen, B-cell receptor [Mus musculus domesticus]	1e-51	72	AAB00850.1
7 SD H	8379 (4481)	immunoglobulin G heavy chain variable region [Homo sapiens]	3e-69	86	AIT38857.1
7 SD L		anti-cytomegalovirus-gB humanized immunoglobulin kappa chain variable region [synthetic construct]	2e-59	85	AAA86295.1

References

1. Dunbar, J. & Deane, C. M. ANARCI: antigen receptor numbering and receptor classification. *Bioinforma.* **32**, 298–300 (2016). DOI 10.1093/bioinformatics/btv552.

2. Honegger, A. & Plückthun, A. Yet Another Numbering Scheme for Immunoglobulin Variable Domains: An Automatic Modeling and Analysis Tool. *J. Mol. Biol.* **309**, 657–670 (2001). DOI 10.1006/jmbi.2001.4662.

3. Chailyan, A., Tramontano, A. & Marcatili, P. A database of immunoglobulins with integrated tools: DIGIT. *Nucleic Acids Res.* **40**, D1230–D1234 (2011). DOI 10.1093/nar/gkr806.

4. Gabbard, J. *et al.* A humanized anti-M2 scFv shows protective in vitro activity against influenza. *Protein Eng. Des. Sel.* **22**, 189–198 (2008). DOI 10.1093/protein/gzn070.

5. Hu, W.-G., Yin, J., Chau, D., Negrych, L. M. & Cherwonogrodzky, J. W. Humanization and Characterization of an Anti-Ricin Neutralization Monoclonal Antibody. *PLoS ONE* **7**, e45595 (2012). DOI 10.1371/journal.pone.0045595.

6. Asano, R. *et al.* Construction and humanization of a functional bispecific EGFRxCD16 diabody using a refolding system. *FEBS J.* **279**, 223–233 (2012). DOI 10.1111/j.1742-4658.2011.08417.x.

7. Jia, X. *et al.* A humanized anti-DLL4 antibody promotes dysfunctional angiogenesis and inhibits breast tumor growth. *Sci. Reports* **6** (2016). DOI 10.1038/srep27985.

8. Shaw, S. *et al.* Discovery and characterization of olokizumab. *mAbs* **6**, 773–781 (2014). DOI 10.4161/mabs.28612.

9. Margreitter, C., Mayrhofer, P., Kunert, R. & Oostenbrink, C. Antibody humanization by molecular dynamics simulations-in-silicoguided selection of critical backmutations. *J. Mol. Recognit.* **29**, 266–275 (2016). DOI 10.1002/jmr.2527.

10. Shembekar, N. *et al.* Humanized antibody neutralizing 2009 pandemic H1N1 virus. *Biotechnol. J.* **9**, 1594–1603 (2014). DOI 10.1002/biot.201400083.

11. Baldassi, C. *et al.* Fast and accurate multivariate Gaussian modeling of protein families: predicting residue contacts and protein-interaction partners. *PloS one* **9**, e92721 (2014).

12. Wang, P. *et al.* A systematic assessment of MHC class II peptide binding predictions and evaluation of a consensus approach. *PLoS computational biology* **4**, e1000048 (2008).

13. Wang, P. *et al.* Peptide binding predictions for HLA DR, DP and DQ molecules. *BMC bioinformatics* **11**, 568 (2010).

14. Gao, S. H., Huang, K., Tu, H. & Adler, A. S. Monoclonal antibody humanness score and its applications. *BMC Biotechnology* **13**, 1–12 (2013). DOI 10.1186/1472-6750-13-55.

15. Altschul, S. F., Gish, W., Miller, W., Myers, E. W. & Lipman, D. J. Basic local alignment search tool. *J. molecular biology* **215**, 403–410 (1990).
